# *In-situ*, In-Memory Stateful Vector Logic Operations based on Voltage Controlled Magnetic Anisotropy

**DOI:** 10.1038/s41598-018-23886-2

**Published:** 2018-04-10

**Authors:** Akhilesh Jaiswal, Amogh Agrawal, Kaushik Roy

**Affiliations:** School of Electrical and Computer Engineering, Purdue University, West Lafayette, IN USA

## Abstract

Recently, the exponential increase in compute requirements demanded by emerging applications like artificial intelligence, Internet of things, *etc*. have rendered the state-of-art von-Neumann machines inefficient in terms of energy and throughput owing to the well-known *von-Neumann bottleneck*. A promising approach to mitigate the bottleneck is to do computations as close to the memory units as possible. One extreme possibility is to do *in-situ* Boolean logic computations by using *stateful* devices. Stateful devices are those that can act both as a compute engine and storage device, simultaneously. We propose such stateful, vector, in-memory operations using voltage controlled magnetic anisotropy (VCMA) effect in magnetic tunnel junctions (MTJ). Our proposal is based on the well known manufacturable 1-transistor - 1-MTJ bit-cell and does not require any modifications in the bit-cell circuit or the magnetic device. Instead, we leverage the very physics of the VCMA effect to enable stateful computations. Specifically, we exploit the voltage asymmetry of the VCMA effect to construct stateful IMP (implication) gate and use the precessional switching dynamics of the VCMA devices to propose a massively parallel NOT operation. Further, we show that other gates like AND, OR, NAND, NOR, NIMP (complement of implication) can be implemented using multi-cycle operations.

## Introduction

The pioneering works of the likes of Charles S. Peirce^1^, Claude Shannon^2^ among others laid the foundation of digital logic design. The fact that few basic digital gates, that form a complete logic basis, can be easily implemented using electronic switches had far reaching implications for the future of digital computing. Indeed, with the invention of transistor switches^3^, digital logic quickly gained ground and has become the workhorse of today’s information processing^4^.

In general, the state-of-art digital processors rely heavily on Boolean gates constituting the *computational unit* which is separate from the *storage unit* consisting of numerous memory cells. This decoupled architecture wherein memory and compute units are physically separated is named after its inventor as the *von-Neumann architecture*^5^. The von-Neumann architecture forms the backbone of almost all the available commercial processors. Despite the tremendous strides made in computing efficiency powered by the von-Neumann machines, it fails to deliver the required speed and efficiency demanded by the recent developments in big-data, artificial intelligence, Internet-of-things (IoT) *etc*^6^. The major limitation associated with the von-Neumann architecture is the so-called *von-Neumann bottleneck*^7^. This bottleneck mainly arises from the limited data transfer rate between the physically decoupled compute and memory units. The frequent *to-and-fro* data transfer between the compute and the memory units, not only limits the overall throughput but also results in large energy overhead associated with each data transfer. In order to mitigate the limitations associated with the von-Neumann bottleneck one promising approach is to enable *in-memory* vector computations^8–10^.

These novel computing paradigms termed as *in-memory* computations aim to implement some (or all) aspects of Boolean logic computations as close to the memory units as possible, thereby avoiding expensive data transfer between the compute and memory units, resulting in higher throughput and better energy-efficiency. Such in-memory computations using conventional silicon based complementary-metal-oxide-semiconductor (CMOS) technology has been demonstrated in ref.^11^. The basic idea behind the in-memory compute mechanism proposed in ref.^11^ is to activate multiple rows of memory-cells and read-out a voltage which is proportional to the desired logic computations. However, silicon technology is itself facing tremendous challenges due to aggressive scaling of the CMOS transistors^12–14^. As such, novel memory technologies like spin based magnetic random access memories (MRAMs)^15,16^, resistive RAMs^17^, phase change materials based memories^18^ are being actively investigated for possible replacement of silicon based technologies. A key benefit of these novel technologies is their *non-volatility*. The non-volatile characteristics of these memory units make them well-suitable for ultra-low leakage applications ultimately increasing the energy-efficiency^19^.

Exploration of in-memory compute designs using such non-volatile technologies are crucial to meet the energy and throughput requirement demanded by the emerging data intensive applications. Spin-transfer-torque MRAM (STT-MRAM) based in-memory Boolean computations have been proposed in refs^20,21^. These in-memory architectures rely on the peripheral read circuits to implement the actual computations. Nevertheless, the peripheral circuits being close to the memory array does provide energy and throughput benefits. However, a major drawback associated with such read circuit based memory computations is the fact that the typical sense margin for STT-MRAM is quite small^22^ and techniques that rely on logic computations based on multi-level sensing of the STT-MRAM bit-cells would inevitably suffer from robustness concerns. Further, the logic computation results are only available when the data is being read from the memory array. This implies if one were to do multiple logic operations which are dependent on the intermediate results, one would require to do a read operation for every logic computation. Thus, each in-memory logic operation is inevitably associated with a memory read operation even for intermediate results, leading to decreased memory throughput and energy-efficiency. As opposed to the aforementioned works which use the memory peripheral circuits to do the actual logic computations, there are other classes of in-memory compute designs that do computations ‘*in-situ*’ using ‘stateful’ memory devices. ‘Stateful’ devices are those wherein the same device acts both as a memory element and compute unit. The well known memristive implication (IMP) logic demonstrated in ref.^23^ is a good example of such stateful computations. However, the limited endurance of memristors in general make these devices unsuitable for on-chip cache or IoT applications that have extreme longevity requirement. Out of all the non-volatile technologies, spin based devices are the only devices that have high switching speed as well as unlimited endurance. Few works on stateful computations using spin devices can be found in refs^24–26^. Specifically, the work presented in ref.^24^ uses a three terminal device exploiting the spin Hall effect and the voltage controlled magnetic anisotropy (VCMA) in spin devices to do stateful computations. However, one of the inputs to these devices is an electrical quantity *i.e*. input charge current. This in turn implies if we were to compute say the vector AND operation on the logic states stored in two separate memory rows, one of the memory rows will have to be read first, then converted into electrical signal (a current in this case) before the actual logic computation can be completed. This requirement of ‘read before compute’ would lead to degraded benefits in throughput and energy. Stateful computations as described in refs^25,26^ do not require the ‘read before compute’ scheme. Besides, works in refs^27–29^ have also shown in-memory compute primitives using spin based magnetic tunnel junctions (MTJs). The compute operations in refs^25–29^ are fundamentally based on the difference in resistance of the MTJ in the parallel and anti-parallel state. Given the fact that the MTJ tunnel magneto-resistance ratio (TMR) is usually small^30^, this leads to highly constrained design space. In the present work, we not only rely on the MTJ TMR but also on the voltage polarity based conditional lowering of the MTJ energy barrier (EB) through the VCMA effect, thereby widening the design space for stateful computations in spin devices. In addition, all the previous works can carry computations corresponding to one single memory-row at a time, we show the possibility of enabling massively parallel *multi-row* NOT and XOR operations (XOR operation described in the Appendix) by exploiting the magnetization dynamics of the MTJs based on the VCMA phenomenon.

In this manuscript, we employ the very physics of voltage controlled magnetic anisotropy to construct *in-situ*, in-memory, stateful computations using a two terminal spin device. Specifically, we use the voltage asymmetry of the VCMA effect to construct IMP (implication) logic and the precessional dynamics of the VCMA switching process to propose a *massively parallel* NOT operation. The key highlights of the present work and its advantages over previous works are as follows:

1. We propose *in-situ*, in-memory stateful IMP vector computations using the voltage asymmetry of the VCMA effect on two terminal magnetic tunnel junctions (MTJs). In addition, we propose a *massively parallel* NOT operation by exploiting the precessional switching dynamics of VCMA based MTJs.
2. Further, the massively parallel behavior of the proposed NOT gate allows multi-cycle computation of other Boolean functions including AND, OR, NAND, NOR, NIMP (complement of IMP), thereby constructing a rich logic functionality embedded within the memory array in a stateful manner.
3. One of the major advantages of the proposed *in-situ*, in-memory stateful vector computations is the fact that we rely on the well known 1 transistor - 1 MTJ bit-cell without making any changes in the magnetic device or the bit-cell circuit. This is turn makes our proposal attractive from manufacturability point of view. Further, as opposed to^20,21^ our logic computations do not rely on complex read operations given the fact that reading MTJ devices in general is a complex circuit problem. In addition, as opposed to the work in ref.^24^, we do not need to represent the logic operands by an electrical input, rather both the logic operands can be stored in the memory array leading to higher throughput.
4. We have developed a detailed device-circuit model comprising of self-consistent magnetization dynamics and electron transport model integrated seamlessly in SPICE environment to study the feasibility of the proposed logic computations.

## VCMA mechanism: Voltage Asymmetry and Precessional Switching

### VCMA mechanism: Voltage asymmetry

**Figure 1:**
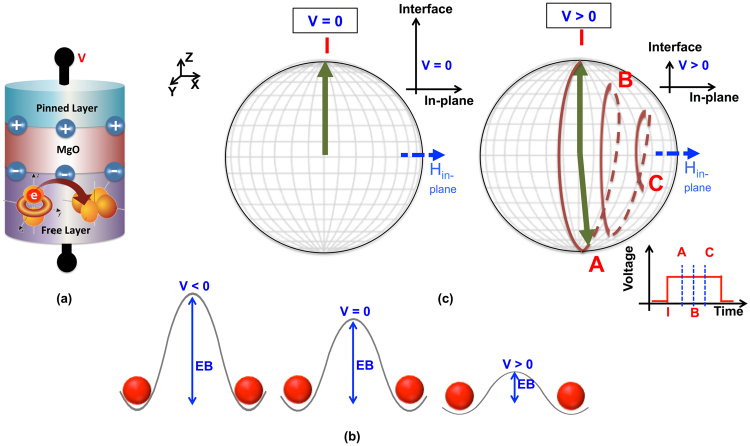
(**a**) A VCMA based MTJ. The MTJ consists of a *pinned layer* and a *free layer* separated by a non-magnetic spacer. When a voltage is applied across the MTJ, there is a redistribution of electrons in the d-orbitals thus making the interface anisotropy sensitive to the applied voltage. (**b**) Schematic representation of the voltage asymmetry of the VCMA based MTJs. When a positive (negative) voltage is applied across the VCMA MTJ the energy barrier (EB) decreases (increases) due to the lowering (enhancement) of the interface anisotropy. Thus, VCMA mechanism makes the MTJ asymmetric with respect to voltage polarity, a positive voltage assists in switching the MTJ whereas a negative voltage makes it much harder to switch the MTJ. (**c**) Figure representing the precessional switching mechanism. When a positive voltage is applied across the MTJ such that the interface anisotropy is sufficiently lowered, the magnetization vector becomes free to precess around the *hard axis* due to the effective in-plane field (*H*_*in*_−_*plane*_). Inset shows the lowering of the interface anisotropy on application of sufficiently high positive voltage (V > 0). While the magnetization vector is precessing around the hard-axis, if the voltage pulse is switched OFF when the magnetization is close to point A, it would slowly dampen towards −z direction, thereby switching the direction of magnetization by 180°. A voltage pulse as a function of time marked with the points corresponding to the state of magnetization vector at a particular time instance is also shown in the figure.

The basic device structure under consideration in this work is the two terminal magnetic tunnel junction (MTJ). An MTJ consists of two nano-magnets separated by an insulating oxide as shown in Fig. 1(a). The MTJ is called a perpendicular MTJ if the magnetization directions of the two nano-magnets are perpendicular to the plane of the nano-magnets. One of the nano-magnets is fixed called the *pinned layer* (PL), while the other nano-magnet can be switched by applying a voltage across the MTJ called the *free layer* (FL). The MTJ has two stable states called the parallel (P) state and the anti-parallel (AP) state. When the magnetization of the two nano-magnets are in the same direction the MTJ is in low resistance P state and *vice-versa*.

Conventionally, the state of the MTJ has been switched using the current induced spin transfer torque (STT) phenomenon^31^. The basic physics associated with the STT phenomenon relies on the fact that a *spin polarized* current passing through the FL exerts a torque on the FL thereby flipping the state of the MTJ from the P to the AP state and *vice-versa*. This exerted torque by the STT mechanism has to be sufficient to overcome the energy barrier (EB) associated with the FL. In perpendicular MTJ, it is the interface anisotropy that creates the required energy barrier between the two stable states of the MTJ. In general, higher the EB, higher is the current required to switch the MTJ. One of the key challenges associated with the STT phenomenon is the high switching current requirement^32^. In order to reduce the current requirement for switching the nano-magnet various voltage driven switching phenomenon are under intense research investigation^33,34^. One of the most promising technique and easy to incorporate in the the two terminal MTJ stack is the voltage controlled magnetic anisotropy (VCMA) effect^33^.

VCMA effect is the phenomenon of being able to modulate the interface anisotropy of the MTJ stack by applying a voltage across the MTJ^35^. Application of an electric field modulates the relative occupancy of the valence d-orbitals, as shown schematically in Fig. 1(a), thereby effectively changing the interface anisotropy^36,37^. Recall, in perpendicular MTJs it is the interface anisotropy that is primarily responsible for creating the required EB. A large EB is required for maintaining the non-volatility of the MTJ devices. However, a large EB also makes it harder to switch the nano-magnets during the write process. VCMA effect allows one to temporarily reduce the EB by reducing the interface anisotropy in response to electric field. The reduced EB makes it easier to switch the nano-magnets, thereby reducing the switching current requirement. On the other hand, if the direction of the electric field is reversed, EB increases due to the VCMA effect making it much more difficult to switch the nano-magnet. This increase or decrease in the EB due to application of an electric voltage across the MTJ is shown schematically in Fig. 1(b). The figure shows that, the VCMA effect makes the MTJ stack *asymmetric* with respect to the voltage polarity. With favorable voltage polarity (pinned layer at higher potential than the free layer) the MTJ can be easily switched while if the voltage polarity is reversed the MTJ would be difficult to switch. In fact, it has been experimentally shown that when the EB is increased by applying a voltage, the MTJ breaks down at sufficiently higher voltages but does not switch^38^. In later section, we would describe how this voltage asymmetry of the VCMA based MTJs would be used to construct stateful IMP logic for vector operations.

### VCMA mechanism: Precessional switching

In addition, the VCMA effect allows for a new switching dynamics, called the *precessional switching*, in contrast to the typical STT based switching phenomenon^39^. The precessional switching dynamics can be understood with respect to Fig. 1(c). Let us assume the magnetization of the FL is initially pointing in +z-direction due to the interface anisotropy that tends to align the magnetization direction perpendicular to the plane of the nano-magnet. As a consequence of the VCMA effect, when a voltage is applied across the MTJ, the interface anisotropy decreases. If the decrease in the interface anisotropy is sufficient, the magnetization would no longer be bound by the interface anisotropy and would be free to deviate from its initial position (+z direction in this case). Now, assume there is a small in-plane field in +x-direction (denoted as *H*_*in−plane*_ in Fig. 1(c)) either due to the shape anisotropy, or such in-plane field can be engineered in the MTJ stack as experimentally demonstrated in ref.^40^. Since, the interface anisotropy has been reduced by voltage application (V > 0 in Fig. 1(c)) and there is an effective field in the +x direction, the magnetization would tend to align itself to the effective field. It would do so by precessing and slowly damping towards the +x direction. This behavior is graphically depicted in Fig. 1(c), where the magnetization initially starts from position ‘I’ and then follows the trajectory marked by points A-B-C on application of electric field across the MTJ (V > 0).

If we turn OFF the applied voltage when the magnetization is at point A in Fig. 1(c), the magnetization would slowly dampen and point in the −z direction due to the interface anisotropy. Thus, by timing the voltage pulse such that magnetization makes a half-cycle around the *hard-axis* (+x in this case), it can be switched by 180°. This switching due to the precession of the magnetization across the hard-axis is called precessional switching. VCMA based precessional switching has several advantages including low energy-requirement and high switching speed^41^. We would later describe how this precessional switching of the VCMA MTJs can be used to construct a massively parallel NOT operation.

## Device Modeling

In this section, we describe the coupled device-circuit simulation model developed for analyzing the proposed stateful logic computations. The model integrates and self-consistently solves the magnetization dynamics and electron transport model in a SPICE platform, enabling a rigorous circuit simulation for evaluating the proposed vector operations.

### Magnetization Dynamics with VCMA

The magnetization vector in a mono-domain nanomagnet follows the dynamics governed by the well-known *Landau-Lifshitz-Gilbert-Slonczewski (LLGS)* equation^42,43^. LLGS equation can be written as follows:

1 $$\frac{\partial \hat{m}}{\partial t}=-|\gamma |\hat{m}\times {\overrightarrow{H}}_{EFF}+\alpha \hat{m}\times \frac{\partial \hat{m}}{\partial t}+\overrightarrow{STT}$$

2 $${\overrightarrow{H}}_{EFF}={\overrightarrow{H}}_{ext}+{\overrightarrow{H}}_{demag}+{\overrightarrow{H}}_{ani}+{\overrightarrow{H}}_{thermal}$$

3 $$\overrightarrow{STT}=|\gamma |\beta (\hat{m}\times (\varepsilon \hat{m}\times \hat{P}+\varepsilon ^{\prime} \hat{P}))$$

where $\hat{m}$ is the unit magnetization vector, *α* is the Gilbert damping constant, *γ* is the gyromagnetic ratio, *H*_*EFF*_ is the effective magnetic field experienced by the nanomagnet and $\overrightarrow{STT}$ is the STT torque acting on the nanomagnet. The first term on the right hand side of Eq. 1 relates to magnetization precession along *H*_*EFF*_ while the second and last terms describe the damping torque and STT, respectively. *H*_*EFF*_ includes an external field (*H*_*ext*_), demagnetization field due to shape anisotropy^44^ (*H*_*demag*_), the interface perpendicular anisotropy field^45^ (*H*_*ani*_) and stochastic field due to thermal noise (*H*_*thermal*_), as described in Eq. 2. The $\overrightarrow{STT}$ torque is expressed in Eq. 3, where *β* is the rate of spin transfer into the MTJ-FL, *ε* is the spin injection efficiency, $\hat{P}$ is the polarization of the incoming spin current and *ε*′ describes the STT field-like torque.

Further, as described earlier, VCMA modulates the interface anisotropy of the MTJ stack in response to an applied voltage. VCMA is thus modeled using a voltage dependent anisotropy constant (*K*_*i*_), which is incorporated in the LLGS equation through *H*_*ani*_, as follows:

4 $${K}_{i}={K}_{i0}-\xi \frac{{V}_{MTJ}}{{t}_{MgO}}$$

5 $${H}_{ani}=\frac{2{K}_{i}{m}_{z}}{{M}_{s}{t}_{FL}}\hat{z}$$

where *ξ* is the VCMA coefficient, *V*_*MTJ*_ is the voltage applied across the MTJ stack, *t*_*Mgo*_ is the spacer oxide thickness, *K*_*i*0_ is the nominal value of anisotropy constant at zero voltage (no VCMA), *M*_*s*_ is saturation magnetization and *t*_*FL*_ is thickness of the FL nanomagnet. The thermal noise was included in the LLGS equation using a thermal field given by Brown’s model^46^ as:

6 $${\overrightarrow{H}}_{thermal}=\overrightarrow{\zeta }\sqrt{\frac{2\alpha {k}_{B}T}{|\gamma |{M}_{S}{\rho }_{mtj}dt}}$$

where $\overrightarrow{\zeta }$ is a vector having components that are Gaussian random variables with zero mean and standard deviation of 1, *ρ*_*mtj*_ is the volume of the nanomagnet, *T* is the ambient temperature, *k*_*B*_ is the Boltzmann’s constant and *dt* is the simulation time step. The device dimensions and other parameters used in our simulations are tabulated in Table 1.

**Table 1:** MTJ parameters used in the simulation model.

Parameters	Value
MTJ Diameter (*W*_*MTJ*_)	40 *nm*
MTJ-FL thickness (*t*_*FL*_)	0.9 *nm*
MTJ-spacer thickness (*t*_*Mgo*_)	1.3 *nm*
MTJ-PL polarization	0.4
Saturation Magnetization (*M*_*S*_)	1257.3 *emu*/*cm*^3^ ^50^
Gilbert Damping Factor (*α*)	0.02
Tunneling Magnetoresistance (TMR)	125%
VCMA coefficient (*ξ*)	3.72*e*−8 *ergV*^−1^/*cm*^49^
Interface Anisotropy, at 0 V (*K*_*i*0_)	1.1*erg*/*cm*^2^
External field (*H*_*ext*_)	$100Oe\hat{y}$

### MTJ Resistance model

**Figure 2:**
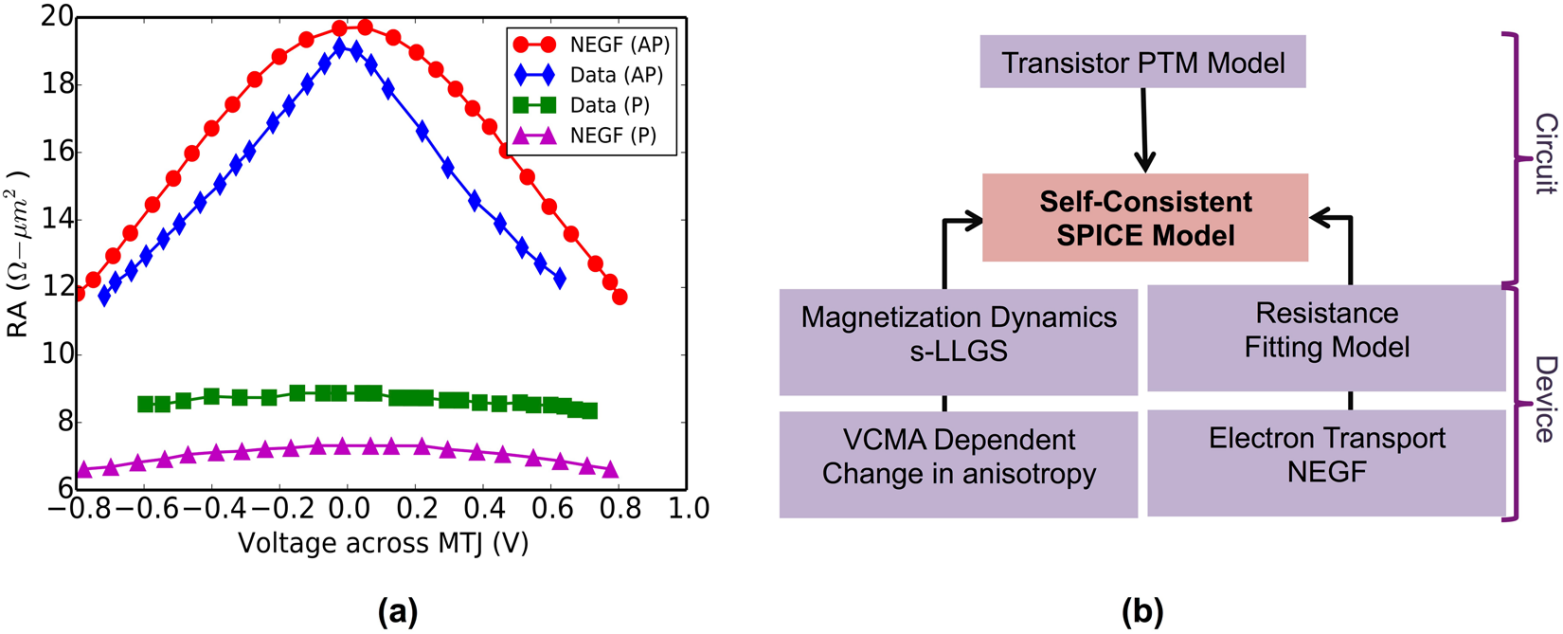
(**a**) The NEGF based MTJ-resistance model^30^ benchmarked against experimental data from^16^. (**b**) A graphical representation of the various components of our self-consistent magnetization dynamics and resistance transport model.

The resistance of the MTJ was modeled using the non-equilibrium Green’s function (NEGF) approach, benchmarked against experimental data from^16^, as illustrated in Fig. 2(a). The details of various equations used in our NEGF model can be found in ref.^30^. Our NEGF model is based on a potential profile wherein a non-magnetic barrier separates two nano-magnets. The non-magnetic barrier is characterized by its energy-barrier while the nano-magnets by their *band-splitting* energy. The results obtained by the NEGF calculations were encapsulated in an analytical fitting model such that the resulting MTJ resistance was modeled as a SPICE compatible voltage dependent resistance.

### Self-Consistent SPICE Compatible Magnetization Dynamics and Resistance Model

A SPICE compatible device-circuit model was developed in Verilog-A for the VCMA-MTJ. The Verilog-A model concurrently solves the LLGS equation, the MTJ resistance model and the associated circuit equations. Predictive transistor models^47^ were used for the access transistors, thus completing the 1-T 1-VCMA MTJ bit-cell model. Figure 2(b) shows graphically the various building blocks associated with our self-consistent device-circuit simulation framework.

## Proposed ***in-situ***, in-memory Stateful Vector Logic Operations

### Stateful vector IMP gates

Let us assume we have two VCMA based MTJs – ‘MTJ-1’ and ‘MTJ-2’ storing two input data bits ‘Bit-1’ and ‘Bit-2’, respectively. We wish to compute the implication (IMP) of bits ‘Bit-1’ and ‘Bit-2’ such that the new value of the MTJ-2 would correspond to the IMP of the original values of bits ‘Bit-1’ and ‘Bit-2’. Further, let us assume that this logic computation has to be done in a ‘stateful’ manner such that the same VCMA MTJs (that function as memory elements storing bits ‘Bit-1’ and ‘Bit-2’) also act as logic computation units.

**Figure 3:**
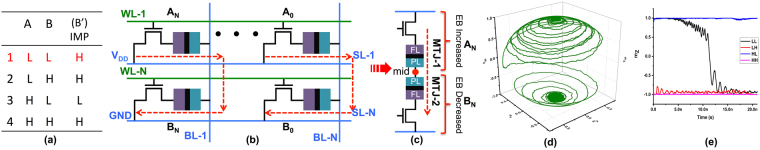
(**a**) The truth table for two input IMP operation. The columns B and B’ are the same except for row 1, highlighted in red. (**b**) The array configuration showing the voltages at various SLs and WLs and the current flow during the stateful computation of the bit-wise IMP operation. (**c**) A simplified circuit showing the voltage divider configuration resulting due to the applied voltages at the SLs and WLs. (**d**) A typical magnetization dynamics during the switching of the MTJ-2 from the P to the AP state, when MTJ-1 is in the P state. Note, this switching dynamics is a typical STT dominated switching, VCMA effect lowers the EB for MTJ-2, thereby allowing the small current flowing through the MTJ-2 to be able to selectively switch the MTJ-2 as desired. (**e**) Figure showing the magnetization component *m*_*z*_ for all the four cases of the truth table shown in (**a**).

In order to understand the proposed stateful computations, let us consider the truth table of a two input IMP gate shown in Fig. 3(a). Note, the first column (A) would physically represent possible states of MTJ-1 and the second column (B) would represent states of MTJ-2. The third column (B’) represents the new state of MTJ-2 after the logic operation has been completed. Interestingly, in Fig. 3(a), column B is same as B’ except for row 1 (highlighted in red). Further, we assume the low digital level (L) is mapped to the P state of the MTJ and high digital level (H) is mapped to the AP state. This implies in order to do the stateful computations, when the operand ‘A’ (MTJ-1) is in the P state and operand ‘B’ (MTJ-2) is also in the P state, the state of MTJ-2 should change from P to AP, thereby mimicking the logic operation corresponding to row 1 of Fig. 3(a). Further, for all other cases since B = B’, the state of the MTJ-2 should not change. Thus, if we can retain the state of MTJ-2 for rows 2, 3, 4 and change the state from P to AP for row 1 we would have effectively accomplished the IMP operation.

Figure 3(b) and (c), illustrates the device-circuit technique to do the aforementioned IMP computation. Let us assume we have two vector input operands ‘A’ and ‘B’. The bits ‘A_0_’ to ‘A_*N*_’ corresponding to the input ‘A’ are stored in upper row of the memory array as shown in Fig. 1(b). Similarly, bits ‘B_0_’ to ‘B_*N*_’ corresponding to the input ‘B’ are stored in lower row of the memory array. In order to do the bit-wise IMP computations for operands ‘A’ and ‘B’ we would activate the corresponding word-lines WL-1 and WL-N. Simultaneously, a voltage V_*DD*_ would be applied to SL-1, while SL-N would be grounded resulting in a current flow as marked by the red arrow in Fig. 3(b). A simplified version of the resulting circuit configuration, considering one column consisting of one bit from the vector operand ‘A’ and corresponding bit from the vector operand ‘B’, is shown in Fig. 3(c).

Figure 3(c) is basically a voltage divider, the voltage at node ‘mid’ depends on the resistance states of MTJ-1 and MTJ-2. Note, in this circuit configuration the pinned-layer of MTJ-1 has a lower voltage than the free-layer, while for MTJ-2 the pinned-layer is at a higher voltage than the free-layer. This in turn implies, with reference to Fig. 1(b), MTJ-1 has a higher energy barrier (EB) while MTJ-2 has a lowered energy barrier owing to the VCMA effect. As such, it is much easier to switch MTJ-2 while the state of MTJ-1 would remain intact due to increase in its EB. Thus, irrespective of the data stored in the two MTJs, MTJ-1 would have a higher EB while MTJ-2 would have a lower EB.

By appropriate choice of *V*_*DD*_ and the MTJ resistances, the circuit in Fig. 3(c) can be designed such that MTJ-2 switches from the P to the AP state only when MTJ-1 is in the P state. A higher voltage at node ‘mid’ (corresponding to the P state of MTJ-1) would imply enhanced lowering of the EB for MTJ-2 allowing the small current flowing through the MTJ-2 to be able to deterministically switch the MTJ-2 from the P to the AP state as desired.

Note, it is due to the lowered EB of the MTJ-2, that the small current flowing through the MTJs can switch the MTJ-2, but not the MTJ-1 (since the EB for MTJ-1 has increased due to its voltage polarity). The current flowing through the MTJ-2 switches its state due to the STT effect, given the fact that the switching current requirement for MTJ-2 has been conditionally (only when MTJ-1 is in the P state) reduced due to the voltage at node ‘mid’. This STT like switching behavior, as shown in Fig. 3(d), is evident from the magnetization dynamics of MTJ-2, simulated using the model described in the previous section. Note, the P to AP switching of the MTJ-2 only when MTJ-1 is in the P state implements both the rows 1 and 3 of the Fig. 3(a). Specifically, when MTJ-1 is in the AP state, voltage at node ‘mid’ is not high enough to sufficiently lower the EB of MTJ-2, thereby retaining its original state corresponding to row 3. However, when MTJ-1 is in the P state, the voltage across MTJ-2 is sufficient to lower the EB of MTJ-2 such that it switches to the AP state corresponding to row 1.

The state for MTJ-2 (corresponding to the column B’ in Fig. 3(a)) for remaining rows 2 and 4 is same as the column B and is the AP state. Further, the current flow direction is such that it always tries to switch MTJ-2 to the AP state. Thus, for rows 2 and 4, MTJ-2 is initially in the AP state, moreover, the current flowing through the MTJ-2 is also trying to switch it to the AP state, thereby the state of MTJ-2 is retained for both rows 2 and 4.

In summary, for implementing the IMP operation, we perform selective switching of the MTJs. This selective switching is a result of the combined effect of the VCMA based lowering of the EB for MTJ-2 and the STT induced torque due to the current flowing through the series connection of the two MTJs. Only when the MTJ-1 is in P state (digital ‘L’) the voltage across the MTJ-2 is high enough to sufficiently lower the EB such that MTJ-2 switches by the STT mechanism from P to AP state (or from digital ‘L’ to ‘H’ state). The switching mechanism is still the STT effect, but the reason why some MTJs switch and others do not is based on the fact that it is the VCMA induced selective lowering of the EB that allows the STT current to be able to switch the MTJs. This selective switching corresponding to all the four cases shown in truth table of Fig. 3(a) is presented in Fig. 3(e). As it can be observed, only for the first row of the truth table the magnetization switches in all other cases it retains it’s original state. As a result, by merely activating WL-1 and WL-2 and applying appropriate voltages on lines SLs, *in-situ* stateful vector IMP operation can be achieved.

### Stateful parallel NOT gates

NOT is a one variable operation, therefore, let us consider a single bit-cell consisting of 1 transistor - 1 VCMA MTJ. In order to reverse the current state of the MTJ we can use the precessional switching dynamics of the VCMA effect. As explained in earlier sections, when sufficient voltage is applied across the VCMA MTJ, the interface anisotropy decreases and in presence of an effective in-plane field the magnetization starts precessing around the hard axis as shown in Fig. 1(c). If the input voltage pulse is clocked such that the magnetization has made a half cycle around the hard axis the direction of magnetization would have been effectively reversed by 180°.

Interestingly, irrespective of whether the initial state of the magnetization vector was pointing in the +z or the −z direction, when a sufficient positive voltage is applied to lower the interface anisotropy, the magnetization would start precessing around the hard-axis. This implies, when the magnetization vector would have completed a half-cycle around the hard-axis, if it initially started from +z direction (−z direction), it would now be pointing closer to the −z direction (+z direction). If the voltage pulse is turned OFF when the magnetization has made a half-cycle around the hard-axis it would effectively have switched by 180°. Therefore, irrespective of the initial state of the MTJ, the magnetization direction would always be reversed if the input voltage pulse is clocked such that the magnetization has only completed a half-cycle around the hard axis.

**Figure 4:**
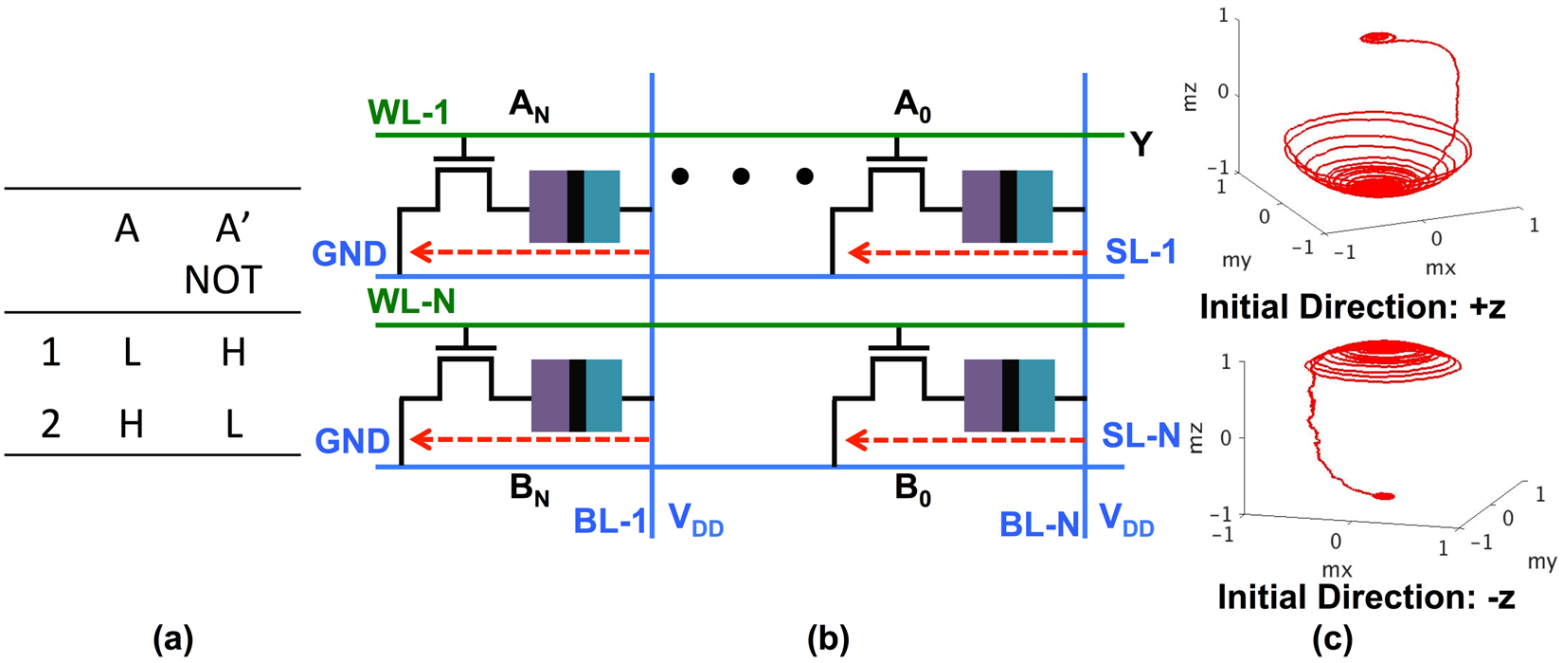
(**a**) The truth table for NOT operation. (**b**) The array configuration showing the voltages at various BLs and WLs and the current flow during the stateful computation of the massively parallel NOT operation. (**c**) A typical magnetization dynamics showing the precessional switching behavior of the VCMA MTJs mimicking the NOT operation. On application of proper voltages, irrespective of the initial state of the magnetization direction (+z or −z), the magnetization vector switches by 180° thereby implementing the desired stateful NOT operation.

This unipolar switching characteristic of the VCMA MTJ, wherein the magnetization always switches by 180° on application of appropriate voltage pulse, can be used to construct a massively parallel vector NOT operation as shown in Fig. 4(b,c). Let us assume we have to do a NOT operation for all the bits corresponding to rows WL-1 and WL-N. Both WL-1 and WL-N would be pulled high to activate the access transistors and proper voltage *V*_*DD*_ needs to be applied to BL-1 through BL-N. This *V*_*DD*_ would be dictated by the VCMA MTJ characteristics such that the magnetization starts precessing around the hard-axis. Usually, the voltage required for VCMA based precessional switching is higher than the voltage requirement for STT-dominated switching^48^. After a predetermined time duration, corresponding to the half cycle precession of the magnetization, the WL and *V*_*DD*_ voltages would be pulled low, thereby reversing the state of all the MTJs connected to both WL-1 and WL-N.

It might be instructive to comment that the switching mechanism during the IMP operation, described in the previous sub-section was STT dominated, the VCMA effect during the IMP operation merely reduced the EB such that the STT current can switch the device. In contrast, for the NOT operation, the switching dynamics is VCMA dominated, that results in precessional switching of the MTJs. The VCMA dominated switching dynamics is also evident from our simulation result shown in Fig. 4(c), which shows a typical magnetization trajectory during the precessional switching based NOT operation. Note, in the upper (lower) part of Fig. 4(c), the magnetization vector starts from +z -axis (−z -axis) and makes approximately a half-cycle around the x-axis before it dampens and consequently settles down in the −z direction (+z direction). Therefore, irrespective of its initial direction, the magnetization vector is always reversed when it completes a half-cycle around the hard axis. The presence of both the STT and VCMA dominated regime in the same MTJ device has been demonstrated experimentally in many works including^48^.

In principle, we can activate all the WLs in the memory array, simultaneously, such that the entire memory array can be flipped in a massively parallel manner. However, in practice the number of WLs that can be simultaneously activated would be limited by the peripheral circuits and the current drivability of the drivers connected to BLs and WLs. Nevertheless, multiple rows can be easily flipped in one cycle resulting in a massively parallel stateful NOT operation. Further, one could argue that precise timing control of the voltage pulses are required for the proper functioning of the NOT operation and given circuit level variations the write-error-rate (WER) for the proposed NOT operation would be exceptionally high. It is worth mentioning, by proper circuit techniques such errors can be mitigated. In fact, as demonstrated in ref.^49^, authors in ref.^49^ were able to obtain WER as low as 1e-14 for precessional switching in VCMA MTJs. A detailed description of the peripheral circuits and write-scheme used for mitigating the WER in precessional switching of VCMA MTJs can be found in ref.^49^. Further, the WER for the AP to the P precessional switching is slightly different from the P to the AP precessional switching. The difference arises due to the existence of the small current flow through the VCMA MTJ favoring one particular switching direction as opposed to the other. However the difference is usually small and has been extensively studied in ref.^41^. In summary, the precessional switching of the VCMA MTJs can be used as a massively parallel NOT operation.

### Other Logic Gates

**Figure 5:**
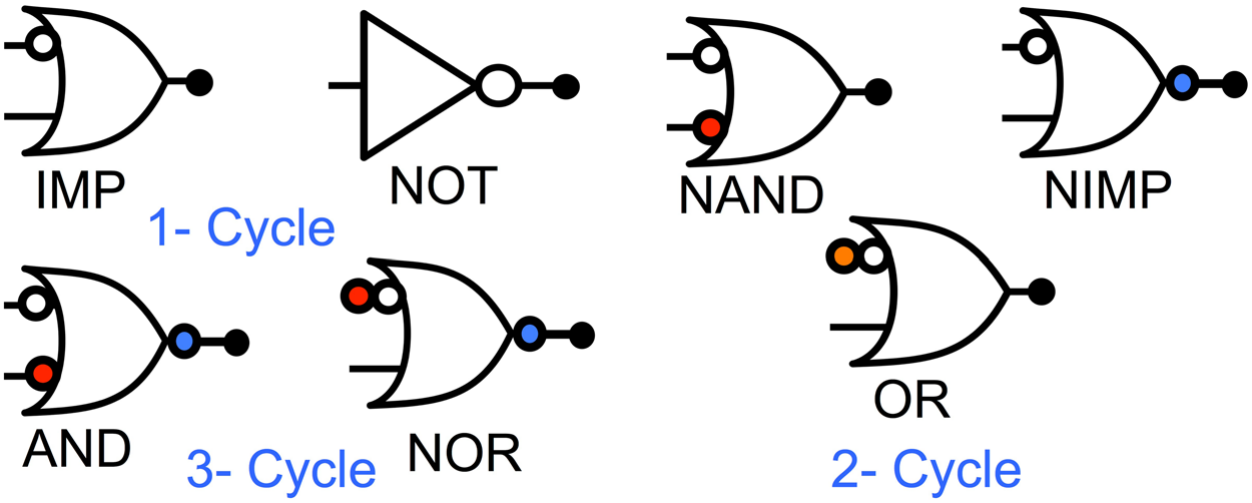
Based on the proposed stateful operations as described in the above sub-sections an IMP and NOT operation can be completed in one cycle, whereas a two cycle operation can implement the NAND, NIMP and OR logic. Similarly, a three cycle operation can be used for the AND and NOR logic computation. For multi-cycle logic, the part of logic highlighted in red can be computed in the first cycle, the part in white can be computed in second cycle, while the part highlighted in blue would be computed in the third cycle.

We have already demonstrated that we can accomplish vector IMP and NOT operations in one cycle. In principle, since the IMP along with NOT operation is a universal gate, the proposed scheme can be used for mapping any arbitrary Boolean computations. However, since NOT is a massively parallel operation, the IMP operation can be combined with the NOT operation to achieve various other basic Boolean gates. For example, as shown in Fig. 5, by using two cycles stateful NAND/OR/NIMP logic operations can be accomplished. Further, if we assume three cycles, stateful AND/NOR operations can be computed using the proposed techniques. Note, as opposed to the stateful IMP logic in memristive crossbars^23^, the present proposal has significant advantages due to the fact that the NOT operation can be achieved in a massively parallel manner that too in the usual 1-transistor - 1-MTJ bit-cell, thereby enabling other stateful logic operation as in NAND/NOR *etc*.

### Normal Memory Read and Write Operations

For the sake of completeness, note that the 1-transistor – 1-VCMA MTJ array can still be used as a conventional memory block. Conventionally, in a usual MRAM array the source line (SL) and the bit line (BL) run parallel to each other while the word line (WL) runs in the orthogonal direction^16^. However, in the present proposal we have the SL and the WL parallel to each other, while the BL is in the orthogonal direction as shown in Figs 3 and 4. For a normal write operation (wherein a particular data has to be stored in an MTJ), we would follow the ‘read before write’ scheme. The ‘read before write’ scheme for precessionally switched VCMA based arrays have been discussed in detail in various previous works including^39,49^. The requirement for such a ‘read before write’ scheme can be understood as follows. Precessional switching can only reverse the direction of magnetization provided the pulse duration is properly chosen so that the magnetization makes approximately a half cycle around the hard axis. This necessitates reading the data stored in the MTJ before a write pulse can be applied. After reading the MTJs, only those MTJs are reversed through the VCMA voltage that are not in the desired state.

Such a write operation can be easily accomplished in the array structure shown in Figs 3 and 4. For performing the write operation, the selected WL would be activated by driving it to a high voltage. The data would first be read by driving the SL to a voltage *V*_*read*_ and sensing the resultant current on the respective BLs. Once the data is read, subsequently only those MTJs would be switched whose original state differs from the data to be written into the MTJs. This can be accomplished by pulling the SL to ground and applying a write voltage of proper duration on those BLs for which the MTJs have to be switched. For all other MTJs, the BL would be grounded, thereby preventing any inadvertent switching.

## Results

In this section, using the comprehensive simulation model described earlier, we evaluate the functionality and performance of the proposed in-memory vector computations. Note, during the process of magnetization switching, the resistance of the MTJ keeps changing which in turn would change the voltage across the MTJ. Therefore, both the STT and the VCMA strength is a function of the instantaneous direction of magnetization. In order to properly capture these effects a self-consistent SPICE model like the one described in the earlier section on device modeling is required as opposed to mixed mode models that solve decoupled LLGS and resistance equations separately.

**Figure 6:**
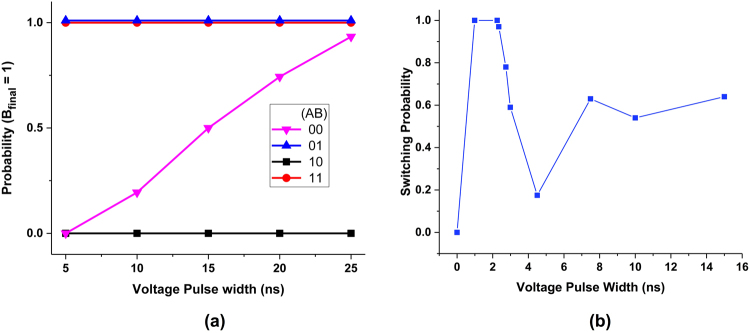
(**a**) Probability of B’s final state being ‘H’ (or digital ‘1’) for the four initial cases of A and B (00, 01, 10, 11) in the vector IMP operation, as a function of the voltage pulse. At a pulse width of ~25 ns, the correct IMP result is obtained. (**b**) Probability of inverting the state of the VCMA MTJ due to precessional switching as a function of the pulse duration. The switching probability peaks at ~2 ns due to the half-cycle rotation of the magnetization dynamics.

The vector IMP operations are performed using the STT-dominated switching of MTJs. In performing an IMP operation on vectors A and B, the current flows from the bit-cells storing bits corresponding to operand A to bit-cells corresponding to operand B, eventually replacing vector *B* with the resulting bit-wise IMP operation (refer to Fig. 3). Also, the negative-VCMA effect on bit-cells storing operand A prevents them from switching their state. Figure 6(a) shows the probability of B’s final state - *which represents the result* - being ‘1’ (or ‘H’ or AP) for the four possible A and B inputs ‘00’, ‘01’, ‘10’ and ‘11’, as a function of the applied voltage pulse width. The simulation is done for various runs in presence of stochastic thermal variations. It can be observed that when the initial state of B is ‘H’ or AP (for inputs ‘01’ and ‘11’), the final state is also AP, irrespective of A’s state. This is because the direction of the current flow restricts B from switching from AP to P state. On the other hand, for the input ‘11’, B never switches its state since the current flowing through the bit-cells in this case is designed to be lower than the critical current required for STT switching, given the fact that the voltage across MTJ-2 is not high enough to sufficiently lower its EB. However, for the input ‘00’, B switches with a probability of ~1, for a voltage pulse width of ~25 ns, thus verifying the functionality and robustness of the bit-wise IMP operation. The average energy consumption per-bit and latency of the IMP operation is tabulated in Table 2.

**Table 2:** Average energy consumption per-bit and latency in the IMP and NOT vector operations.

Vector operation	Average Energy	Latency	*V*_*DD*_
IMP	1.22pJ	25 ns	1.7 V
NOT	0.067pJ	2 ns	0.8 V

While IMP uses STT-dominated switching, NOT operation is primarily VCMA-dominated. As described earlier, the magnetization starts precessing along the hard-axis when a sufficient voltage is applied across the MTJ (see Fig. 4(c)). Note that the *V*_*DD*_ for the NOT operation is specifically chosen, so as to ensure VCMA-dominated precessional dynamics. Figure 6(b) shows the switching probability as a function of voltage pulse width, in presence of thermal variations. The switching probability shows an oscillatory behavior since the final state of the MTJ depends on the magnetization vector direction at the instant when the voltage is turned off. Such oscillating switching probability is typical for precessionally switched magnets. When the magnetization makes a half-cycle of precession (~2 ns) around the hard-axis, a switching probability close to 1 is achieved, thus confirming the expected functionality for the NOT operation. The presented figure is for the P to the AP switching, similar oscillating probability was also obtained for the AP to P switching. Note that the NOT operation is massively parallel. Even multiple vectors can be inverted simultaneously, by activating the corresponding WLs and SLs of the bit-cells. Table 2 enumerates the energy consumption per-bit and latency of the NOT operation.

Before we conclude the manuscript, it is informative to mention that the present proposal relies on the VCMA as well as the STT effect for implementing the ‘stateful’ computations. The key material parameters that are crucial for proper functioning of the proposed schemes are the VCMA co-efficient and the TMR ratio. Higher the VCMA co-efficient better is the change in the energy barrier in response to applied voltage. Similarly, higher the TMR more is the resistance difference between the parallel and the anti-parallel state of the MTJ and better is the control of the MTJ resistance on the STT current flowing through the series MTJs of Fig. 3(c). As such, material stacks that exhibit higher TMR and VCMA co-efficient would be better suited for the proposed ‘stateful’ gates. The typical material parameters we used in our simulations are mentioned in Table 1.

## Conclusion

The conventional von-Neumann computing architecture fails to deliver the required energy and throughput efficiency for emerging data intensive applications like artificial intelligence, IoT *etc*. Enabling in-memory computations is being hailed by the research community as a promising technique with a potential to go beyond the von-Neumann computing model. The basic idea driving such in-memory computations is to enable logic computations as close to the memory unit as possible. An extreme possibility is to do logic computations *‘in-situ*’ in stateful manner, wherein the same device acts like a storage element as well as logic computation engine. In this manuscript, we have proposed

**Figure 7:**
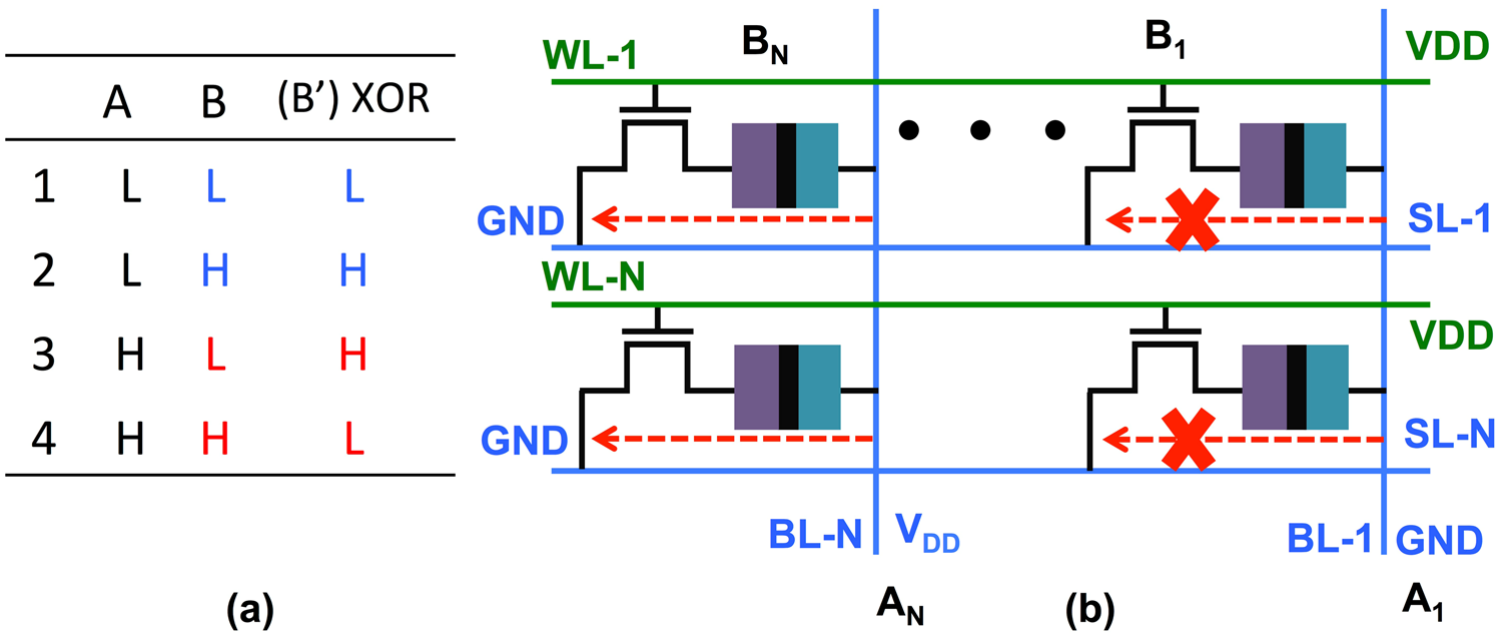
(**a**) A truth table for XOR gate. The logic output B’ retains its original value when the operand A is ‘L’, whereas if the operand A is ‘H’, the new value for B’ is the complement of its original value B. (**b**) Figure shows the array structure used for implementing the XOR operation. The voltages on BLs represent the bits corresponding to the operand A, while the data stored in the MTJs represent the bits corresponding to the operand B. The values in the MTJs are inverted conditionally only if the bits corresponding to the operand A are ‘H’ *i.e*. only if the respective SLs are pulled high. Note, in the example shown, the bit value for A_1_ is ‘L’, as such, BL-1 is kept low. Therefore, no current flows through the column corresponding to BL-1 and hence the bits corresponding to BL-1 consume no energy.

*in-situ*, in-memory Boolean stateful computations by leveraging the very physics of voltage controlled magnetic anisotropy in MTJs. The voltage asymmetry of VCMA based MTJs has been used to propose a stateful IMP operation, while the precessional switching dynamics has been exploited for constructing a massively parallel NOT operation. Further, various other gates including AND, OR, NAND, NOR, NIMP can be easily computed using multi-cycle operations. Our results have been verified by a detailed self-consistent magnetization dynamics and resistance model. In addition, the present proposal does not require any changes in the basic magnetic device or the bit-cell circuit, thereby making our proposal feasible from manufacturability point of view.

## Electronic supplementary material

Supplementary Information
