# Supplementary material for: In-situ, In-Memory Stateful Vector Logic Operations based on Voltage Controlled Magnetic Anisotropy
Source: Sci Rep. 2018 Apr 10;8:5738. doi: 10.1038/s41598-018-23886-2 (PMC5893602; doi:10.1038/s41598-018-23886-2)
Supplement: Supplementary file 1 — Supplementary Information [file 41598_2018_23886_MOESM1_ESM.pdf]

Appendix

Here we describe how one can implement stateful XOR operation using the precessional switching dynamics of the VCMA mechanism. The reason we have included the XOR operation in the appendix is due to the fact that unlike the IMP and NOT operation proposed in the manuscript, the XOR operation requires representation of one of the operands as an electrical input *i.e.* one of the operands is represented by the voltage on the bit-line (BL).

This implies if we were to compute the XOR of two vector operands stored in two different rows of the memory array, one of the rows will have to read first, then converted into an electrical input (a voltage in this case) and applied to the BL before the XOR operation can be completed. This results in a requirement of 'read before compute' as opposed to the IMP and NOT operations. However, an interesting possibility is the fact that the XOR operation exploits the precessional switching dynamics and therefore, has the potential of enabling massively parallel XOR operations similar to the NOT operation.

In order to understand the functionality of the stateful XOR operation, let us consider the truth table of the XOR gate as shown in Fig. 7(a). The key observation with respect to the truth table is that the operand B retains its original value when the operand A is 'L' (highlighted in blue), whereas when the operand A is 'H' the state of the operand B has to be inverted (highlighted in red). This implies the XOR operation can be seen as conditional NOT operation, wherein the operand B is inverted only when the operand A is 'H'.

Figure 7

(a) A truth table for XOR gate. The logic output B' retains its original value when the operand A is 'L', whereas if the operand A is 'H', the new value for B' is the complement of its original value B. (b) Figure shows the array structure used for implementing the XOR operation. The voltages on BLs represent the bits corresponding to the operand A, while the data stored in the MTJs represent the bits corresponding to the operand B. The values in the MTJs are inverted conditionally only if the bits corresponding to the operand A are 'H' i.e. only if the respective SLs are pulled high. Note, in the example shown, the bit value for A1 is 'L', as such, BL-1 is kept low. Therefore, no current flows through the column corresponding to BL-1 and hence the bits corresponding to BL-1 consume no energy.

	A	B	(B') XOR
1	L	L	L
2	L	H	H
3	H	L	H
4	H	H	L

(a)

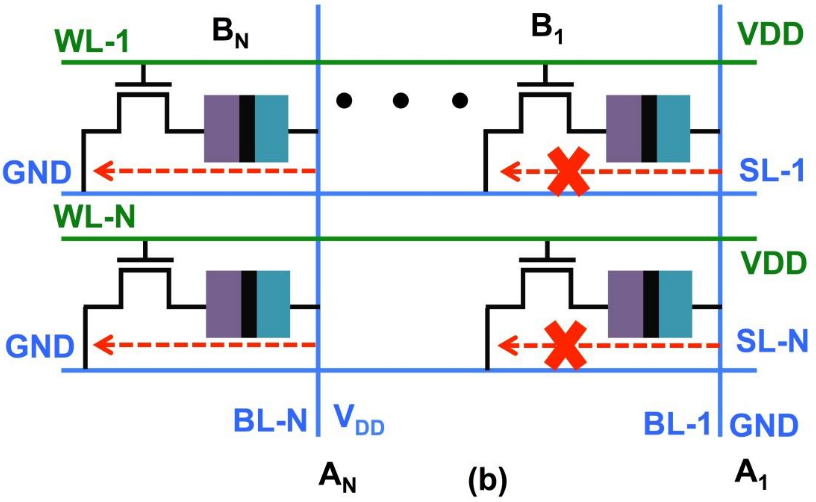

We have already seen in previous sections that the precessional switching dynamics of the VCMA mechanism can be used to perform the NOT operation. Based on such precessional mechanism the proposed bit-wise, stateful, parallel XOR operation can be performed as shown in Fig. 7(b). The operand A is represented as the voltages on the lines BL. For example, if the Nth bit of the vector operand A is A_N = 'H', BL-N would be pulled up to V_{DD} and if A_1 = 'L', BL-1 would remain at 0 volts. The row WL-1 that is supposed to store the vector operand B would then be activated by pulling WL-1 to a high voltage. By ensuring the WL-1 is ON only for a time duration such that the pulse width corresponds to the half-cycle of the magnetization vector, the bits of operand B can be conditionally inverted based on whether the corresponding bit of operand A was 'H' or 'L', thereby completing the XOR operation.

A major benefit of the proposed stateful XOR operation is the fact that we apply a non-zero voltage to the BL only if the corresponding bit of the operand A is 'H'. As such, for those cases where the corresponding bit of the operand A is 'L', the concerned bit-cells consume no energy as both the SL and the BL for those bits are at zero volts. Statistically, this would reduce the energy consumption by almost 50%. Given the extensive use of the XOR operation in many compute applications and the fact that implementing XOR using CMOS transistors is expensive in terms of both energy and area, the present proposal potentially paves the way for low energy and low area XOR in-memory computations. Another benefit of the proposed XOR operation is the possibility of doing a massively parallel operation

similar to the NOT operation. Suppose, the operand A is an encryption key that has to be XORed with all the data stored in multiple rows of the memory array. In principle, all the WLs can be simultaneously activated, such that all the bits in the corresponding rows flip conditionally based on the voltages at respective SLs, thereby completing the XOR operation for multiple rows in a single cycle. The energy consumption for the proposed XOR operation per bit is same as the NOT operation except the fact that in 50% cases when the bits of operand A are zeros, no energy would be consumed.
